# Numerical Analysis of H-PDLC Using the Split-Field Finite-Difference Time-Domain Method

**DOI:** 10.3390/polym10050465

**Published:** 2018-04-24

**Authors:** Sergio Bleda, Jorge Francés, Sergi Gallego, Andrés Márquez, Cristian Neipp, Inmaculada Pascual, Augusto Beléndez

**Affiliations:** 1 Instituto Universitario de Física Aplicada a las Ciencias y las Tecnologías, Universidad de Alicante, P.O. Box 99, E-03080 Alicante, Spain; sergio.bleda@ua.es (S.B.); sergi.gallego@ua.es (S.G.); andres.marquez@ua.es (A.M.); cristian@ua.es (C.N.); pascual@ua.es (I.P.); a.belendez@ua.es (A.B.); 2 Departamento de Física, Ingeniería de Sistemas y Teoría de la Señal, Universidad de Alicante, P.O. Box 99, E-03080 Alicante, Spain; 3 Departamento de Óptica, Anatomía y Farmacología, Universidad de Alicante, P.O. Box 99, E-03080 Alicante, Spain

**Keywords:** H-PDLC, FDTD, diffraction efficiency, LD director distribution, Monte Carlo, 42.40.Eq, 47.11.Bc, 42.70.Jk, 78A10

## Abstract

In this work, an accurate numerical modeling of the diffraction properties of transmission holographic polymer dispersed liquid crystal (H-PDLC) gratings is presented. The method considers ellipsoid geometry-based liquid crystal (LC) droplets with random properties regarding size and location across the H-PLDC layer and also the non-homogeneous orientation of the LC director within the droplet. The direction of the LC director inside the droplets can be varied to reproduce the effects of the external voltage applied in H-PDLC-based gratings. From the LC director distribution in the droplet, the permittivity tensor is defined, which establishes the optical anisotropy of the media, and it is used for numerically solving the light propagation through the system. In this work, the split-field finite-difference time-domain method (SF-FDTD) is applied. This method is suited for accurately analyzing periodic media, and it considers spatial and time discretisation of Maxwell’s equations. The scheme proposed here is used to investigate the influence on the diffraction properties of H-PDLC as a function of the droplets size and the bulk fraction of LC dispersed material.

## 1. Introduction

Holographic polymer dispersed liquid crystal (H-PDLC) gratings are formed by the interference of light on a mixture of liquid crystal (LC), monomer and dye (which shows sensitivity to light). During the recording process of H-PDLC gratings, a separation between polymer-rich and LC-rich areas is produced in the so-called photo-polymerization-induced phase separation process (PIPS) [1]. In this process, the polymerization generates areas in which the LC is concentrated, forming droplets. This LC domain has the same period as that of the recording interference pattern. The processes of recording H-PDLC are well described in [1,2]. A user can modify the LC orientation controlling an external field, setting up an electrically-switchable hologram. Moreover, H-PDLCs have additional well-known properties such as birefringence and optical anisotropy, amongst others. As a result of all these properties, H-PDLCs are of great interest due to the possibility of applying them to many optical systems such as electrically-switchable [3,4] and tunable photonic devices [5], wavelength filters [6], 3D displays [7,8], holographic waveguides [9], optical memories [10] and photonic crystals [11,12,13].

H-PDLCs regarding diffractive optics behave as Bragg gratings. However, the morphological arrangement of the different droplets, the size and the ratio between the area of the LC and the polymer are not usually considered in the approaches used for analyzing this kind of system. There are some theories to analyze this kind of problem. It is worth noting some of them such as the shaped-droplet model based on Montemezzani’s coupled wave theory [14,15], Wu’s model of droplet axis reorientation [16] and the effective medium theory by Sutherland in [17]. Kubytsky et al. [18] combined the developments in [17] with the Monte Carlo simulation for predicting the director profile within the droplet.

Some works regarding the application of finite-difference approaches towards the simulation of LCs can be found in the literature. Wang et al. [3] applied the standard FDTD method to the analysis of LC-based switchable Bragg gratings. A specific application of H-PDLC and FDTD was published by Kubytsky et al. [2] that applied the approach to a regular array of cylindrical LC droplets. In this work, the LC director projection out of the plane of incidence is considered zero. However, in real LC droplets, this condition may not be fulfilled. The main problem derived from applying finite-difference schemes to periodic media is related to the spatial discretization of Maxwell’s equations. This discretisation forces a limit on the extent of the simulation grid. Thus, a finite plane of incidence is defined instead of the infinite plane of incidence needed in periodic media. To avoid this problem, periodic boundary conditions (PBCs) should be applied. However, PBCs show low accuracy for oblique angle plane wave sources in the standard FDTD scheme due to the phase variation across a unit period of the structure and the time-domain nature of the method. In both works previously mentioned [2,3], the drawbacks related to the application of the standard FDTD formulation to periodic media without periodic boundary conditions are shown [19].

The split-field FDTD (SF-FDTD) scheme avoids the drawbacks previously addressed by using a set of field transformation techniques that permits an accurate simulation of plane waves under an oblique angle of incidence [20]. SF-FDTD has been the focus of some researchers that improved the formalisms to make them useful for different situations such as anisotropic media [21,22], birefringent and dichroic media [23], non-linear materials [24,25] and high-performance computing (HPC) [25,26,27].

Here, we present the results of the simulation of the light propagation through the H-PDLC composed by a set of random droplets concerning shape, size and location embedded in a polymeric solid matrix. To the best of our knowledge, this is the first numerical solution that models the random behavior of the sample creation regarding size, allocation and non-homogeneous orientation of the LC director within the LC droplet. The authors consider that this approach is quite similar to the actual generation process of a real H-PDLC grating. The formulation chosen for the numerical simulation permits accurate simulation of arbitrary anisotropic media [21]. The LC director profile is directly predicted using the Monte Carlo (MC) and Metropolis simulation [2,18,28] for each spatial cell in the SF-FDTD grid. Hence, from the orientation of the LC director, the different components of the permittivity tensor are derived and used to update the electromagnetic fields as a function of time and space. The results show that this model sets up a reliable numerical solution for predicting the behavior of H-PDLC.

## 2. Model Description

**Figure 1:**
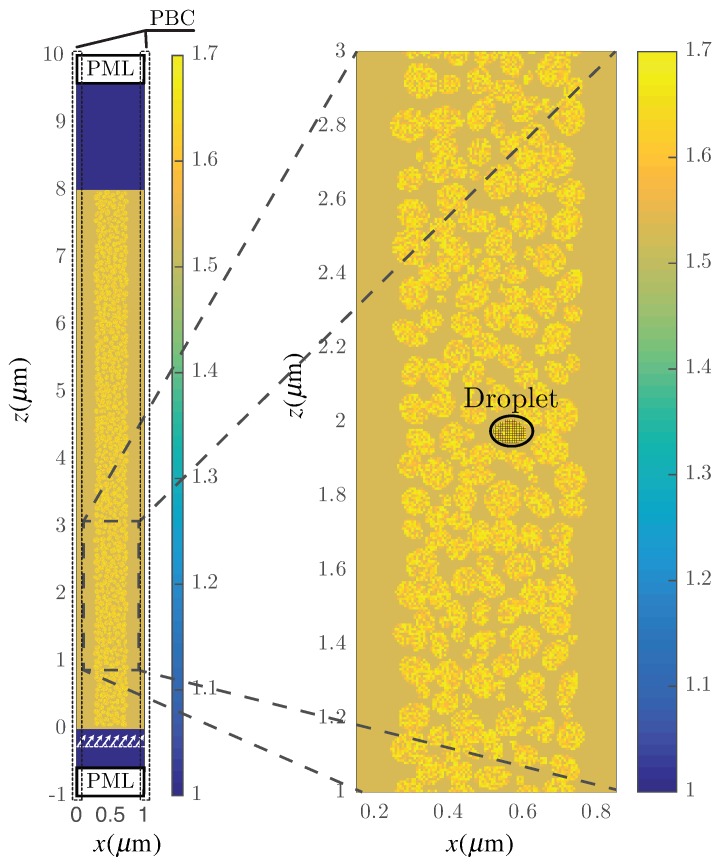
Geometry of the computational domain. The color map represents the refractive index of the media.

The considered setup for modeling two-dimensional H-PDLC structures is based on a spatial discretisation of the medium that permits solving Maxwell’s equations using SF-FDTD. Figure 1 shows the computational domain, where one period of the H-PDLC structure is represented. Periodicity is along the *x*-axis, and the thickness of the layer is along *z*-axis. Specifically, Figure 1 illustrates an H-PDLC grating with a thickness *d* = 8 µm. The plane wave is generated in a virtual interface (see the white arrows at the bottom of Figure 1) between the media and the perfectly matched layer (PML) considered in both the upper and the bottom edges of the grid [29]. PML uses a non-physical lossy medium to limit the extent of the computational domain in the non-periodical direction. Instead of considering the electric and magnetic fields the new field variables P and Q are discretized in the SF-FDTD equations. More precisely, the following variables are considered:

(1) $$\begin{array}{ccc} P & = & Ee^{jk_{x}x}, \end{array}$$

(2) $$\begin{array}{ccc} Q & = & c\mu_{0}He^{jk_{x}x}, \end{array}$$

where *c* is the speed of light for free space, $\mu_{0}$ is the magnetic permeability in a vacuum, $k_{x}=\left(\omega /c\right)\sin\theta_{\mathrm{inc}}$, with $\theta_{\mathrm{inc}}$ being the angle of incidence of the plane waves and ω the angular frequency. E and H are the electric and magnetic field amplitudes, respectively. Materials in this paper are assumed to work in the linear regime, i.e., only three dielectric constants ($\epsilon_{1,2,3}$) and the Euler angles (α,β,γ) are necessary to specify the full tensor description (ϵ):

(3) $$\epsilon =\left[\begin{array}{ccc} \epsilon_{xx} & \epsilon_{xy} & \epsilon_{xz}\\ \epsilon_{yx} & \epsilon_{yy} & \epsilon_{yz}\\ \epsilon_{zx} & \epsilon_{zy} & \epsilon_{zz} \end{array}\right]=T^{-1}\left(\alpha ,\beta ,\gamma \right)\left[\begin{array}{ccc} \epsilon_{1} & 0 & 0\\ 0 & \epsilon_{2} & 0\\ 0 & 0 & \epsilon_{3} \end{array}\right]T\left(\alpha ,\beta ,\gamma \right),$$

where $\epsilon_{i}$, with i=1,2,3 the relative dielectric constants corresponding to the principal axes of the coordinate system. T is the transformation matrix that is fully detailed in [21].

Substituting (1) and (2) into Maxwell’s equations, a set of differential equations is generated that can be solved through the application of a finite-difference approach for both time and spatial derivatives. The reader can find specific information about the implementation of this method in the literature, e.g., the works of Roden et al. [20] established the bases of the approach, and Oh et al. [21] extended the SF-FDTD formulation for the periodic anisotropic structures. The authors have fully implemented this method to include acceleration techniques based on parallel computing and HPC solutions [25].

**Figure 2:**
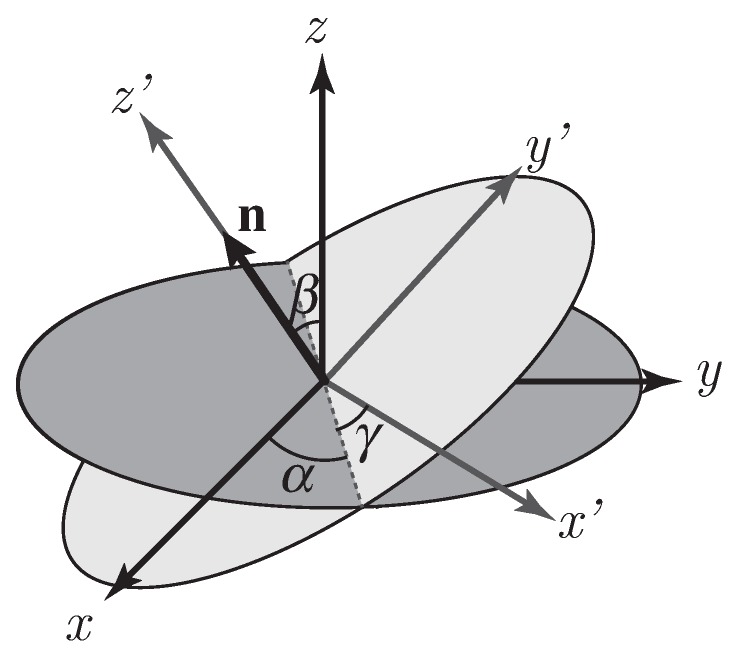
Diagram scheme with the Euler angles. The LC director vector is identified with n.

The director distribution inside the LC droplet is numerically simulated to find the relative dielectric permittivity tensor of LC. The relationship between the angles α, β and γ shown in (3) and the LC director vector n can be identified in Figure 2. Lebhohl and Lasher defined in [30] a simple model of nematic LC. This model assumes that particles are treated as interaction spins with a variable orientation, but fixed concerning the position. The MC method with the Metropolis algorithm is in charge of minimizing the free energy [2,18] defined in Equation (4).

(4) $$E=-\xi \sum_{<i,j>}P_{2}\left(\cos\theta_{ij}\right),$$

with $P_{2}\left(x\right)=1/2\left(3x^{2}-1\right)$ (second rank Legendre polynomial), $\cos\theta_{ij}=n_{i}\cdot n_{j}$; where $n_{i}$ denotes the unit vector giving the orientation of the spin located at the *i*-th grid cell, which in our present context corresponds to the LC-director in the *i*-th grid cell. It is worth noting that even when 2D SF-FDTD scheme is considered, the orientation of the spin is not restricted to a two-dimensional plane. ξ is the maximum interaction energy, and <i,j> indicates adjacent neighbors only. This algorithm starts considering a random initial orientation of each LC director vector of each grid cell in a droplet. The energy $E_{n}$ is then calculated. A random orientation is performed, and the energy $E_{n+1}$ of the new state is then obtained. The energies of both configurations are compared, and if the new state produces a reduction of the free energy, then it is retained. If the new state does not reduce the free energy ($E_{n}<E_{p}$), it will be retained with a probability $P=e^{(E_{n+1}-E_{n})/kT}$. The parameter kT is the product of the Boltzmann constant, *k*, and the temperature, *T*. This probability controls how fast the LC directors tend to be aligned along their long axes. The same procedure is then applied to each grid cell that defines the different droplets. The repetition of this process yields the equilibrium distribution [30]. The final orientation of the LC director within different droplets does not depend on the starting configuration. Other works reproduce the orientation of LC with physical models that are more complex such as finite element methods or variational calculus [31,32,33]. However, the authors consider that this model shows a good accuracy with a reasonable computational complexity for simulating the realistic behavior of the electromagnetic field interaction with the LC.

To be accurate, each single LC droplet must be represented using several grid points. In this work, we are considering the average size for an LC droplet in the range of 50–100 nm and using 170 grid cells on average to represent each LC droplet. The level of detail for each droplet can be seen in Figure 1. Precisely, in this example, 367 droplets with a random size and form have been placed randomly inside the LC region. Figure 1 shows a zoom of an area of the LC region in which the different droplets can be seen in more detail. As an example, one single droplet has been emphasized in the figure to illustrate the degree of precision of the spatial discretisation. The droplet marked in the figure is composed of exactly 100 squared cells.

To reduce the computational needs, some strategies based on non-uniform grid spaces can be used. However, because the libraries implemented here take advantage of HPC solutions, a uniform grid scheme provides accurate results with competitive time costs per simulation.

**Figure 3:**
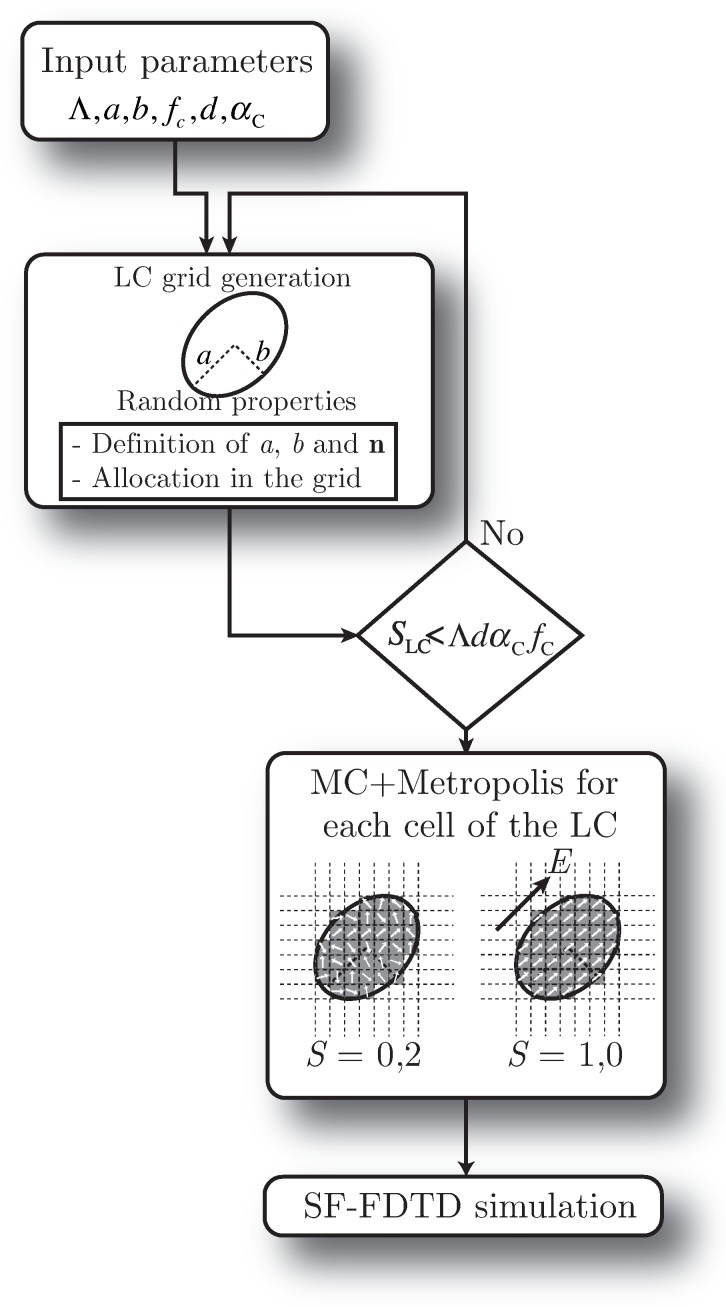
Diagram scheme of the method for simulating H-PDLC. SF, split-field.

Figure 3 shows the scheme of the proposed algorithm. Firstly, an initial set of parameters is defined and introduced in the algorithm. Some of these parameters are related to the spatial configuration of the diffraction grating, e.g., the period of the structure Λ, the thickness of the grating *d*, $\alpha_{C}$ the fraction of the LC stripe in the period and $f_{C}$ the bulk fraction of the dispersed material. There are some additional parameters related to the microstructure of the droplets such as the major and minor sizes of the LC droplet axes *a* and *b*, respectively. It is worth mentioning that the algorithm uses *a* and *b* as starting guesses to set up each droplet. However, the actual sizes of the different droplets are slightly modulated with a random pattern dealing with a set of aleatory droplets regarding size and form (see Figure 1).

The allocation of the droplets inside the LC region is also random, and the algorithm avoids superposition of droplets. The algorithm initially computes the final area that will be filled with LC. If an ideal case is considered, all the LC and the polymer would be allocated in different regions of the grating (total phase separation). Thus, the LC area would be $\Lambda \alpha_{C}d$. However, since the LC is allocated in droplets in a solid polymeric matrix, the actual area filled with LC is slightly lower. Actually, the method establishes an upper limit known as $\Lambda d\alpha_{C}f_{C}$. Consequently, after allocating each droplet inside the LC region, the overall surface of the droplet is updated. If the total surface of the LC droplets is larger than $\Lambda d\alpha_{C}f_{C}$, then the creation of the sample finishes. If there is enough space to place more droplets, the algorithm continues until the latter condition is fulfilled.

The next step is to establish the orientation of the LC director vector. This process is performed once for each droplet and also for each grid cell within each droplet. The final direction of each cell composing the droplet is computed using the MC method according to the Metropolis algorithm [2,30]. The parameter *S* represents the degree of order inside each droplet.

(5) $$S=\frac{1}{2}\left(3\cos{\left(\theta \right)}^{2}-1\right),$$

where θ is the polar angle from the *z*-axis that defines the LC director vector n. The value of S is in the range of zero for extreme disorder and one for a homogeneously parallel alignment [34]. The final configuration of the droplets can be defined by the user employing the MC Metropolis algorithm that provides the angles that define the LC director axis vector of the droplet. These angles are those defined in Equation (3) and represented in Figure 2. Here, the γ angle is neglected since it represents the rotation spin of the vector and has no physical effects on the propagation of light. The MC procedure updates angles α and β. Let us note that when an external electric field is applied across the H-PDLC cell, i.e., along the *z*-axis, the LC molecules tend to align their LC-director with the electric field due to their electric dipole moment [1]. This alignment means that larger values of *S* will emulate the application of a larger external field. Hence, a proportional relationship between *S* and the external voltage can be assumed.

Finally, SF-FDTD performs the update of the electromagnetic fields as a function of time and space in the grid. In all cases, the illumination wavelength is 633 nm, and the grid density is 100 cells by wavelength; thus, the spatial resolution is Δu = 0.633 nm. The time resolution is a function of the angle of incidence and is computed through the Frederich–Levy–Courant condition [20,21,29].

## 3. Results and Discussion

To corroborate the accuracy of the method, the analysis of two gratings has been performed. The parameters of these gratings are summarized in Table 1. Some of these parameters have been previously defined in Section 2. The refractive index of the polymer is represented by $n_{\mathrm{pol}}$, and $n_{⊥}$ and $n_{∥}$ are the LC ordinary and extraordinary refractive indexes, respectively. Grating #1 has the same parameters as those used by Kubytskyi et al. [2], and it is the basis of our validation. Grating #2 is an excellent example of an over-modulated grating, and the values of the parameters are directly related to real materials available by the authors [35].

**Figure 4:**
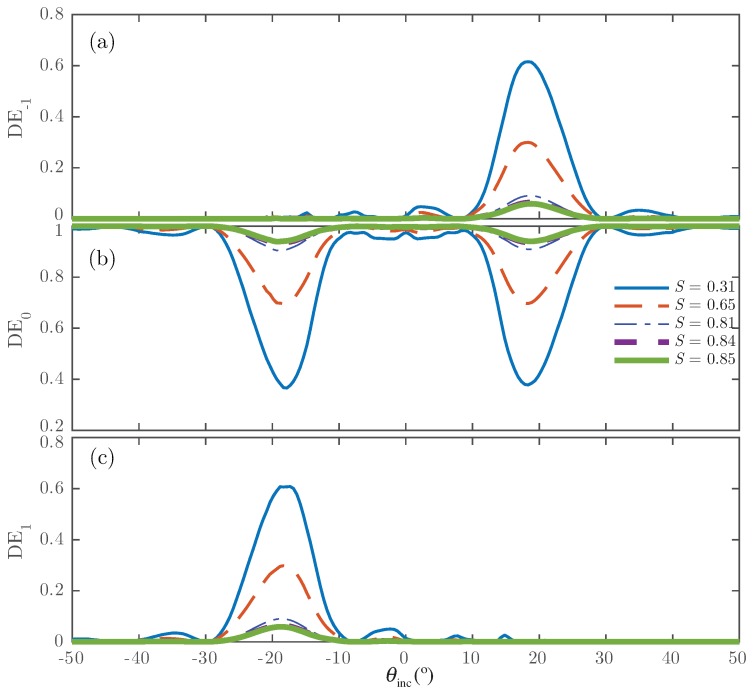
Diffraction efficiency as a function of the angle of incidence for different *S* parameter for Grating #1: (**a**) −1st-order; (**b**) 0th-order; (**c**) 1st-order.

Figure 4 shows for Grating #1 the normalized output diffraction efficiencies as a function of the angle of incidence and different orientations of the director vector of the droplets. Because of the random orientation of the director profile of the LC droplets, the maximum values in the diffraction efficiency for the ±1st-orders occur. For this case, the effective refractive index perceived by the electric field in the *y* component is larger than the $n_{\mathrm{pol}}$; thus, the grating modulation also grows. Increasing *S* indicates the application of a higher external electric field and leads to reorientation of the LC director inside the droplets. Because of this, the dielectric permittivity of the droplets tends towards the value given by the polymer ($n_{\mathrm{pol}}$). In this situation, the modulation of the permittivity tensor becomes small, and the diffraction efficiencies are accordingly reduced.

It is worth mentioning that as *S* tends to zero, the droplet becomes optically isotropic [34] with the average refractive index given by:

(6) $$n=\frac{n_{∥}+2n_{⊥}}{3}.$$

**Figure 5:**
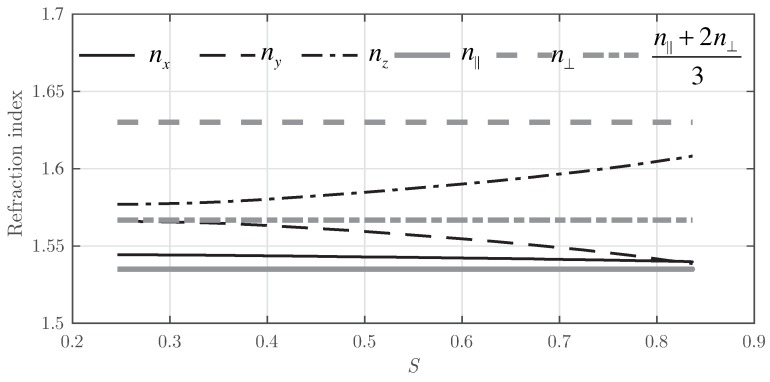
Evolution of the refractive index for the *x*, *y* and *z* components as a function of *S* for Grating #1.

This phenomenon can be corroborated in Figure 5. Initially, the refractive index of the three principal spatial coordinates is closer to the asymptotic value given by Equation (6). As *S* grows, the average refractive index in each component tends to be closer to the refractive index given by the final oriented LC droplet. Thus, $n_{z}$ tends to $n_{⊥}$, whereas $n_{x}$ and $n_{y}$ tend to $n_{∥}$.

**Figure 6:**
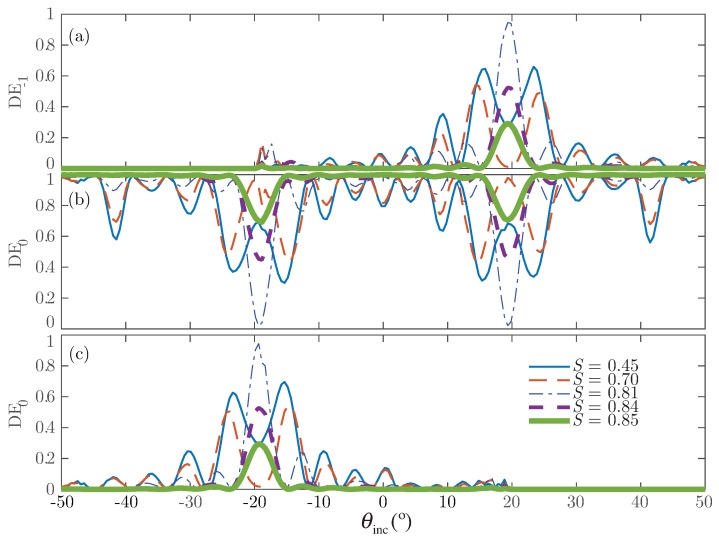
Diffraction efficiency as a function of the angle of incidence for different *S* parameter for Grating #2: (**a**) −1st-order; (**b**) 0th-order; (**c**) 1st-order.

Figure 6 shows the results for Grating #2. This grating is a volume grating with a large thickness. This configuration produces an over-modulation that can be identified in all orders.

The results shown in Figure 4 are similar to those shown by Kubytsky et al. in [2], thus validating the scheme proposed here. In addition, the results represented in Figure 6 are close to those given by the authors in [36] and for Ellaban et al. in [37]. The curves are consistent and show the over-modulated behavior commonly perceived in some holographic applications [36,37]. These results demonstrate the potential of the scheme, since it offers the possibility to analyze the complex structure formed in an H-PDLC accurately and realistically.

### 3.1. Reproducibility Analysis

To check the reproducibility, the analysis carried out is based on generating, using the scheme illustrated in Figure 3, two new different samples of the Grating #1, whose parameters are detailed in Table 1. Since the exact location, size and shape of the different droplets are randomly chosen for each sample, the diffraction efficiencies of these samples are compared to determine whether there are significant differences between samples.

**Figure 7:**
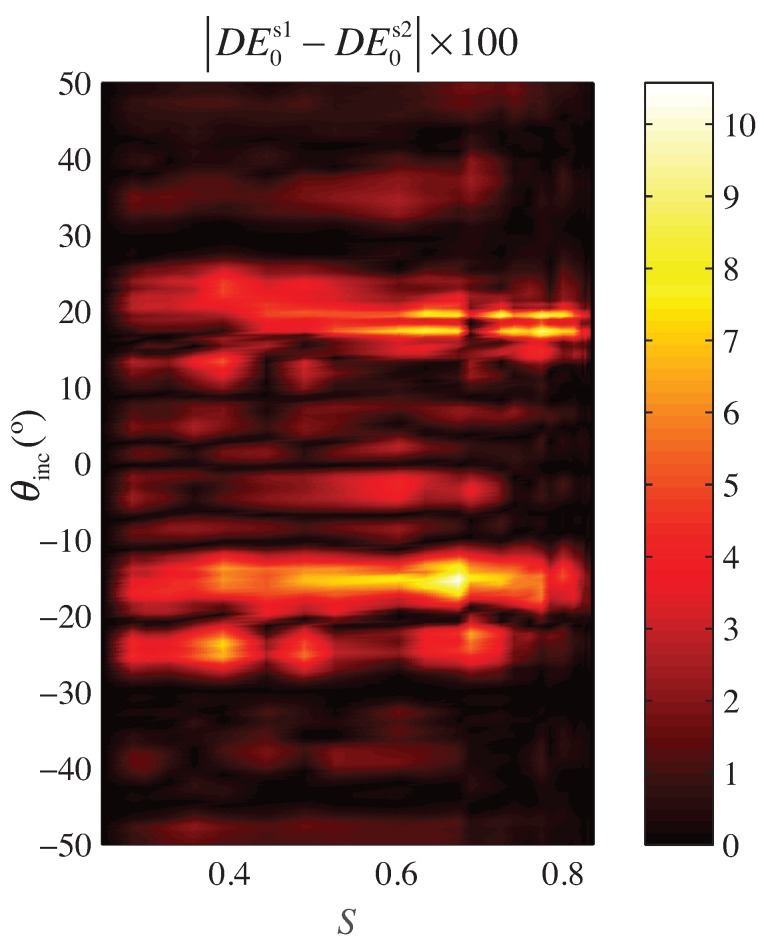
Error between two samples of Grating #1. The color map shows the modulus of the error of the diffraction efficiency (zeroth-order) between samples expressed in percentage.

Owing to the results shown in Figure 7, it can be concluded that the differences between samples are low enough to consider them almost similar. The higher values of the error are produced in the Bragg angle in which the ±1st-orders have more energy (≈±19°). In this situation, the error is lower than the 10%. The mean error analysis of the different samples permits one to ensure that the reproducibility of the experiment is close to the 98.9%. Therefore, it can be concluded that the method provides a consistent set of samples and that the random distribution of the droplets for this setup does not affect in a significant manner the repeatability and reproducibility of the results presented.

### 3.2. Analysis of the Size of the Droplets and Fill Factor

**Figure 8:**
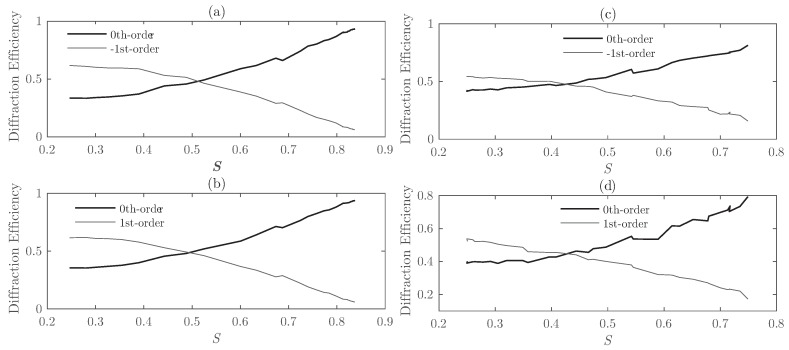
For Grating #1, diffraction efficiency as a function of the *S* parameter for $\theta_{\mathrm{inc}}$ = 18.34° (Bragg angle). Graphs (**a**,**c**) compare the 0th-order with the −1st-order and (**b**,**d**) the 0th-order and the 1st-order. (**a**,**b**) represent the results for droplets between 50 and 100 nm on average; (**c**,**d**) represent the results for droplets between 75 and 150 nm.

In this section, the size of the droplets has been slightly modified to identify its effect on the overall response of the setup proposed. More precisely, the average size of LC droplets has been increased by 50%, from 50–100 nm to 75–150 nm for Grating #1. Figure 8 shows the diffraction efficiency as a function of *S* for the Bragg angle. Figure 8a shows that for S≈0.5, the zeroth- and first-orders share the same values, whereas, for the same *S* value and using larger LC droplet sizes, the diffraction efficiency of the first-order is considerably lower than for the zeroth-order. Figure 8b reveals that the effect of increasing the size of the LC droplets deals with a more irregular and chaotic curve. Increasing the droplet size also produces a slight displacement of the cross point between the diffraction efficiency curves of the zeroth- and first-orders. This issue reveals that smaller droplets provide higher diffraction efficiencies across a broader range of reduced values of *S*.

**Figure 9:**
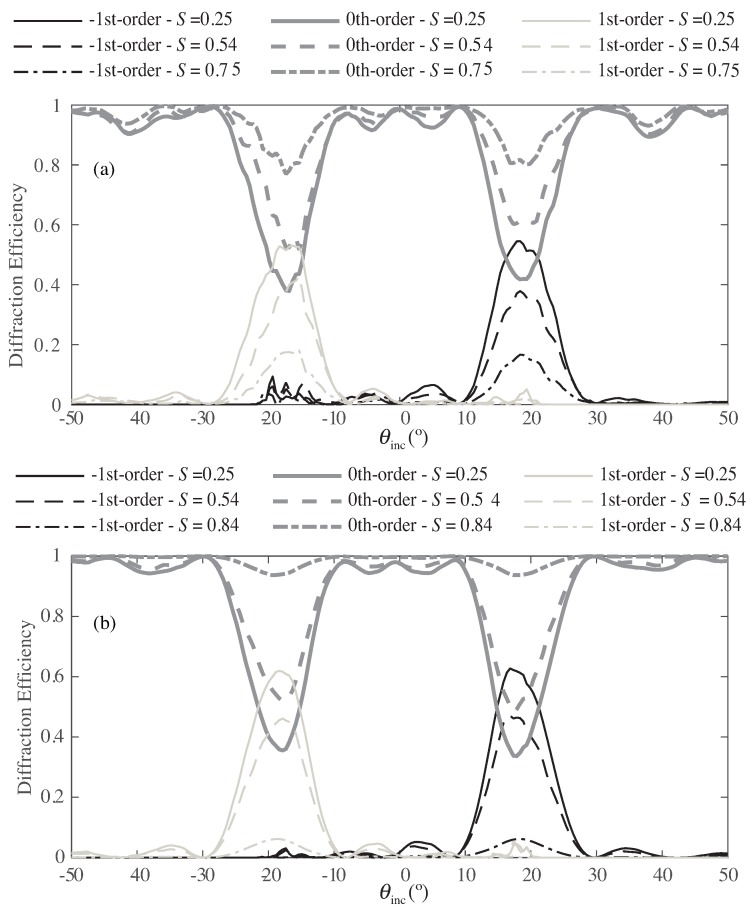
Diffraction efficiencies for the zeroth- and ±1st-orders as a function of θ for different values of *S* for Grating #1: (**a**) Droplets sizes 75–150 nm; (**b**) droplet sizes 50–100 nm.

Figure 9 shows the diffraction efficiencies for the zeroth- and ±1-orders as a function of the angle of incidence and for different orientations of the LC director for Grating #1. For larger droplet sizes, the curves become less homogeneous. Again, a small reduction of the diffraction efficiency with the same LC orientation in both cases can be appreciated. The cause of these effects is the light-induced scattering due to the increased droplet size. Even though it is necessary to address, the size difference is not significant enough to be close to the PDLC regime [38,39].

**Figure 10:**
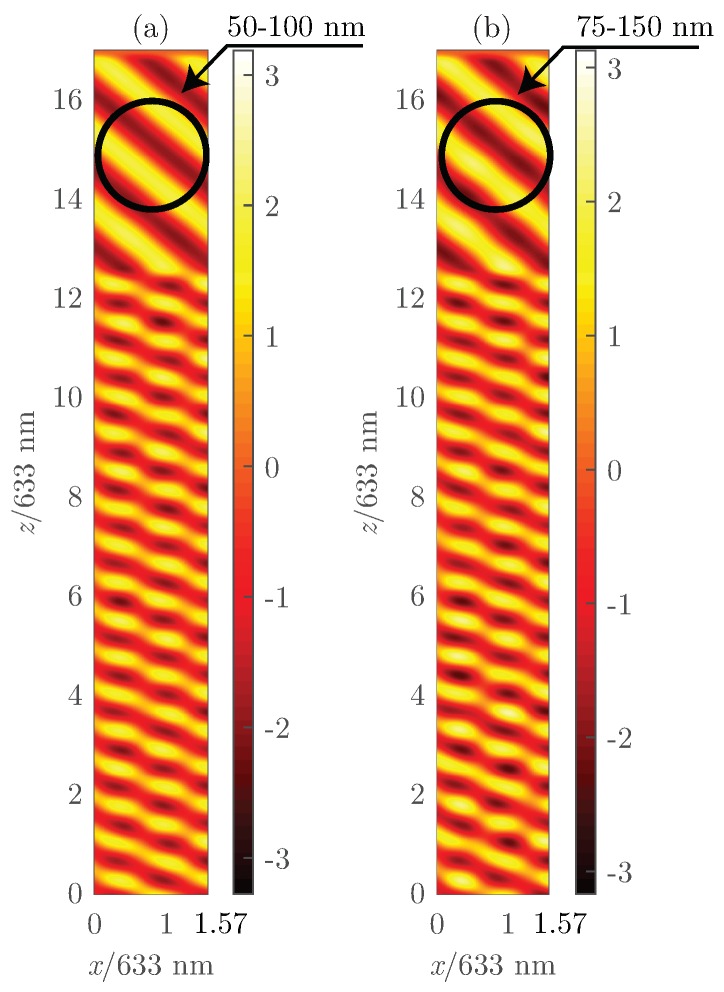
Field distribution of the $E_{y}$ component as a function of space for $\theta_{\mathrm{inc}}$ = 50° and S=0.74: (**a**) size of the droplets between 50 and 100 nm; (**b**) size of the droplets between 75 and 150 nm.

To clearly see the consequence of larger LC size, Figure 10 shows a snapshot of the $E_{y}$ component field for the same grating (Grating #1) with the same illumination properties, but with different LC droplet sizes. Both *x*- and *y*-axes are normalized to the input wavelength (633 nm). Hence, the grating can be identified between z=0 and $z=d/\lambda_{0}=12.3$. The *x* axis takes values between zero and $x=\Lambda /\lambda_{0}=1.57$, which belongs to the spatial periodicity of the structure. The scattering effect produced by the LC droplets can be easily identified comparing the output pattern of the fields for the grating with smaller droplet sizes (Figure 10a) and larger droplet sizes (Figure 10b).

**Figure 11:**
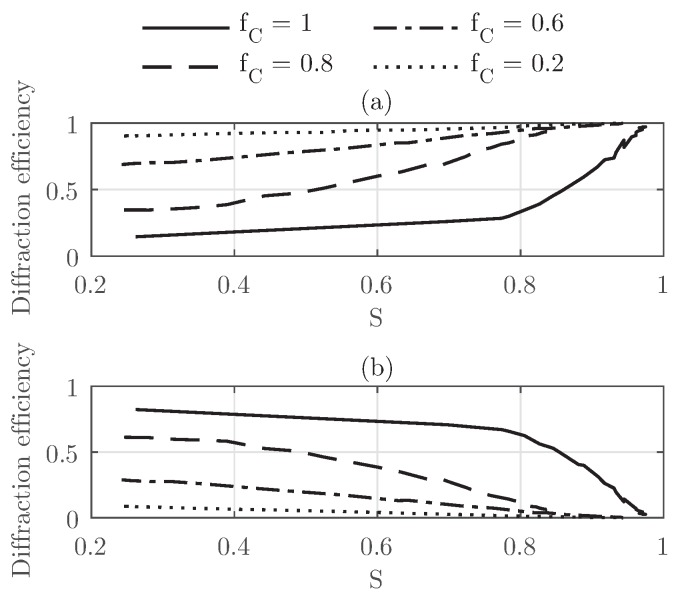
Diffraction efficiencies at the Bragg angle as a function of *S* for Grating #1 for different values of $f_{C}$ and angle of incidence $\theta_{\mathrm{inc}}$ = 18.34° (**a**) zeroth-order; (**b**) −1st-order.

The fill factor $f_{C}$ has also been analyzed, and the results are summarized in Figure 11. The diffraction efficiencies as a function of *S* are represented for the zeroth-order and the −1st-order for different values of $f_{C}$. From this set of curves, two evident behaviors as a function of the $f_{C}$ can be perceived. The first one can be easily identified as a linear dependence of the diffraction efficiency with the orientation of the LC director *S* for the cases $f_{C}=0.2,0.6$. As the fill factor increases the slope of the curves, they become steeper. Secondary to this linear trend for lower fill factors is the effect seen for the highest fill factor considered ($f_{C}$ = 1). For this specific case, as *S* decreases, the amplitude of the diffraction efficiency for the −1st-order diminishes. From the curve in Figure 11b, the point of a diminished slope can be easily identified close to S≈0.8. For both orders, the influence of the orientation of the LC droplet director becomes linear for S<0.8 and $f_{C}=1$. For $f_{C}=0.8$, the behavior is somewhere in between the linear case of the lower cases for the fill factor and the behavior shown for $f_{C}=1$. For this case, a change in the curve similar to that found for $f_{C}$ = 1 can be identified; however, it is more difficult to identify since the whole behavior of the curve tends to be more homogeneous.

## 4. Conclusions

This work presents a realistic and accurate model for analyzing H-PLDC structures. This model is based on three different parts. Firstly is the creation of the H-PDLC sample that is composed of a set of randomly-placed and -sized LC droplets inside the LC-rich region of the grating. Secondly, the MC with the Metropolis approach is used to induce a reorientation for the LC droplet directors. The director profile of each LC droplet is discretized in a finite grid, and the permittivity tensor is computed and considered in the SF-FDTD simulation for the time-updating of the electromagnetic fields. Here, the SF-FDTD is considered since it is one of the best approaches for accurately simulating optical periodic media. The results show that this setup is a powerful tool for providing an accurate estimation of a manufactured H-PDLC structure since the reproducibility of the results is close to 98.9%. The user can analyze the effect of the LC droplet sizes, their allocation and also the orientation of the director profile in LC.

The effect of increasing the size of the droplets deals with a scattering that reduces the diffraction efficiency by a significant amount. The impact of the fill factor has also been considered, and we observe that the influence of the reorientation of the LC directors in the droplets is linear for a range between $f_{C}=0-0.6$, whereas for larger values of $f_{C}$, the diffraction efficiency presents a decreased slope as the order parameter is reduced. For the specific case of $f_{C}=0.8$, the diffraction efficiency of the −1st-order falls dramatically for S>0.8. A complementary behavior is also identified for the zeroth-order in all cases.

The authors are considering including the effects of losses in the simulation to analyze more precisely the influence of this parameter in thick gratings. Moreover, the next step is to add a sort of optimization process to be able to identify the physical parameters given a measured response of a real sample. This methodology would help in a significant manner the design of an optimization process for applications of H-PDLC in holography and diffractive optics.

## Figures and Tables

**Table 1:** Parameters of the gratings considered.

	Grating #1	Grating #2
Λ (µm)	1	0.96
*d* (µm)	8	18
$\alpha_{C}$	0.5	0.5
$f_{C}$	0.8	0.8
$n_{⊥}$	1.54	1.77
$n_{∥}$	1.63	1.52
$n_{\mathrm{pol}}$	1.53	1.52

## References

[1] Bunning T.J., Natarajan L.V., Tondiglia V.P., Sutherland R.L. (2000). Holographic Polymer-Dispersed Liquid Crystals (H-PDLCs). Ann. Rev. Mater. Sci..

[2] Kubytskyi V., Reshetnyak V. (2007). Finite-difference time-domain method calculation of light propagation through H-PDLC. Semiconduct. Phys. Quantum Electron. Optoelectron..

[3] Wang B., Wang X., Bos P.J. (2004). Finite-difference time-domain calculations of a liquid-crystal-based switchable Bragg grating. J. Opt. Soc. Am. A.

[4] Crawford G.P. (2003). Electrically Switchable Bragg Gratings. Opt. Photon. News.

[5] Sutherland R.L., Tondiglia V.P., Natarajan L.V., Bunning T.J. (2001). Evolution of anisotropic reflection gratings formed in holographic polymer-dispersed liquid crystals. Appl. Phys. Lett..

[6] Jakubiak R., Bunning T., Vaia R., Natarajan L., Tondiglia V. (2003). Electrically Switchable, One-Dimensional Polymeric Resonators from Holographic Photopolymerization: A New Approach for Active Photonic Bandgap Materials. Adv. Mater..

[7] Wang K., Zheng J., Gao H., Lu F., Sun L., Yin S., Zhuang S. (2015). Tri-color composite volume H-PDLC grating and its application to 3D color autostereoscopic display. Opt. Express.

[8] Lloret T., Navarro-Fuster V., Ramírez M., Ortuño M., Neipp C., Beléndez A., Pascual I. (2018). Holographic Lenses in an Environment-Friendly Photopolymer. Polymers.

[9] Neipp C., Francés J., Martínez F., Fernández R., Alvarez M., Bleda S., Ortuño M., Gallego S. (2017). Optimization of Photopolymer Materials for the Fabrication of a Holographic Waveguide. Polymers.

[10] Ogiwara A., Watanabe M., Moriwaki R. (2013). Formation of temperature dependable holographic memory using holographic polymer-dispersed liquid crystal. Opt. Lett..

[11] Tondiglia V., Natarajan L., Sutherland R., Tomlin D., Bunning T. (2002). Holographic Formation of Electro-Optical Polymer–Liquid Crystal Photonic Crystals. Adv. Mater..

[12] Escuti M.J., Qi J., Crawford G.P. (2003). Tunable face-centered-cubic photonic crystal formed in holographic polymer dispersed liquid crystals. Opt. Lett..

[13] Escuti M., Crawford G. (2004). Mesoscale Three Dimensional Lattices Formed in Polymer Dispersed Liquid Crystals: A Diamond-Like Face Centered Cubic. Mol. Cryst. Liq. Cryst..

[14] Montemezzani G., Zgonik M. (1997). Light diffraction at mixed phase and absorption gratings in anisotropic media for arbitrary geometries. Phys. Rev. E.

[15] Sutherland R.L. (2002). Polarization and switching properties of holographic polymer-dispersed liquid-crystal gratings. I. Theoretical model. J. Opt. Soc. Am. B.

[16] Wu B.G., Erdmann J.H., Doane J.W. (1989). Response times and voltages for PDLC light shutters. Liq. Cryst..

[17] Sutherland R.L., Tondiglia V.P., Natarajan L.V., Lloyd P.F., Bunning T.J. (2006). Coherent diffraction and random scattering in thiol-ene–based holographic polymer-dispersed liquid crystal reflection gratings. J. Appl. Phys..

[18] Kubytskyi V., Reshetnyak V., Galstian T. (2006). Electrically Controllable Diffraction Efficiency of H-PDLC Film Composed of Ellipsoidal Liquid Crystal Droplets. Mol. Cryst. Liq. Cryst..

[19] Francés J., Bleda S., Neipp C., Márquez A., Pascual I., Beléndez A. (2013). Performance analysis of the {FDTD} method applied to holographic volume gratings: Multi-core {CPU} versus {GPU} computing. Comput. Phys. Commun..

[20] Roden J., Gedney S., Kesler M., Maloney J., Harms P. (1998). Time-domain analysis of periodic structures at oblique incidence: Orthogonal and nonorthogonal FDTD implementations. IEEE Trans. Microwave Theory Tech..

[21] Oh C., Escuti M.J. (2006). Time-domain analysis of periodic anisotropic media at oblique incidence: An efficient FDTD implementation. Opt. Express.

[22] Oh C., Escuti M.J. (2007). Numerical analysis of polarization gratings using the finite-difference time-domain method. Phys. Rev. A.

[23] Miskiewicz M.N., Bowen P.T., Escuti M.J. (2012). Efficient 3D FDTD analysis of arbitrary birefringent and dichroic media with obliquely incident sources. Proc. SPIE.

[24] Shahmansouri A., Rashidian B. (2011). Comprehensive three-dimensional split-field finite-difference time-domain method for analysis of periodic plasmonic nanostructures: Near- and far-field formulation. JOSA B.

[25] Francés J., Bleda S., Álvarez M.L., Martínez F.J., Márquez A., Neipp C., Beléndez A. (2013). Acceleration of split-field finite difference time-domain method for anisotropic media by means of graphics processing unit computing. Opt. Eng..

[26] Shahmansouri A., Rashidian B. (2012). GPU implementation of split-field finite-difference time-domain method for Drude-Lorentz dispersive media. Prog. Electromagn. Res..

[27] Francés J., Bleda S., Bej S., Tervo J., Navarro-Fuster V., Fenoll S., Martínez-Gaurdiola F.J., Neipp C. (2016). Efficient split field FDTD analysis of third-order nonlinear materials in two-dimensionally periodic media. Proc. SPIE.

[28] Kubitskiy V., Reshetnyak V., Galstian T. (2005). Electric Field Control of Diffraction Efficiency in Holographic Polymer Dispersed Liquid Crystal. Mol. Cryst. Liq. Cryst..

[29] Taflove A., Hagness S.C. (2005). Computational Electrodynamics: The Finite-Difference Time-Domain Method.

[30] Lebwohl P.A., Lasher G. (1972). Nematic-liquid-crystal orde—A Monte Carlo calculation. Phys. Rev. A.

[31] Vanbrabant P.J.M., Beeckman J., Neyts K., James R., Fernandez F.A. (2009). A finite element beam propagation method for simulation of liquid crystal devices. Opt. Express.

[32] Beeckman J., James R., Fernández F.A., De Cort W., Vanbrabant P.J.M., Neyts K. (2009). Calculation of fully anisotropic liquid crystal waveguide modes. J. Lightwave Technol..

[33] Abdulhalim I., Menashe D. (2010). Approximate analytic solutions for the director profile of homogeneously aligned nematic liquid crystals. Liq. Cryst..

[34] Yeh P., Gu C. (1999). Optics of Liquid Crystal Displays.

[35] Ortuño M., Riquelme M., Gallego S., Márquez A., Pascual I., Beléndez A. (2013). Overmodulation Control in the Optimization of a H-PDLC Device with Ethyl Eosin as Dye. Int. J. Polym. Sci..

[36] Neipp C., Álvarez M.L., Gallego S., Ortuño M., Sheridan J.D., Pascual I., Beléndez A. (2004). Angular responses of the first diffracted order in over-modulated volume diffraction gratings. J. Mod. Opt..

[37] Ellaban M.A., Glavan G., Klepp J., Fally M. (2017). A Comprehensive Study of Photorefractive Properties in Poly(ethylene glycol) Dimethacrylate—Ionic Liquid Composites. Materials.

[38] Drzaic P.S. (1995). Droplet density, droplet size, and wavelength effects in PDLC light scattering. Mol. Cryst. Liq. Cryst..

[39] Montgomery G.P., West J.L., Tamura-Lis W. (1991). Light scattering from polymer-dispersed liquid crystal films: Droplet size effects. J. Appl. Phys..

